# A network analysis of anxiety, depressive, and psychotic symptoms and functioning in children and adolescents at clinical high risk for psychosis

**DOI:** 10.3389/fpsyt.2022.1016154

**Published:** 2022-10-28

**Authors:** Gabriele Lo Buglio, Maria Pontillo, Erika Cerasti, Andrea Polari, Arianna Schiano Lomoriello, Stefano Vicari, Vittorio Lingiardi, Tommaso Boldrini, Marco Solmi

**Affiliations:** ^1^Department of Dynamic and Clinical Psychology, and Health Studies, Faculty of Medicine and Psychology, Sapienza University of Rome, Rome, Italy; ^2^Child Psychiatry Unit, Department of Neuroscience Bambino Gesù Children’s Hospital, IRCCS, Rome, Italy; ^3^Istituto Nazionale di Statistica (Istat), Rome, Italy; ^4^Orygen Specialist Programs, Melbourne, Australia; ^5^Centre for Youth Mental Health, The University of Melbourne, Parkville, VIC, Australia; ^6^Section for Cognitive Systems, DTU Compute, Technical University of Denmark, Kongens Lyngby, Denmark; ^7^Department of Life Science and Public Health, Catholic University of the Sacred Heart, Rome, Italy; ^8^Department of Developmental Psychology and Socialization, University of Padua, Padua, Italy; ^9^School of Epidemiology and Public Health, Faculty of Medicine, University of Ottawa, Ottawa, ON, Canada; ^10^Department of Psychiatry, University of Ottawa, Ottawa, ON, Canada; ^11^Department of Mental Health, The Ottawa Hospital, Ottawa, ON, Canada; ^12^Ottawa Hospital Research Institute (OHRI) Clinical Epidemiology Program University of Ottawa, Ottawa, ON, Canada; ^13^Department of Child and Adolescent Psychiatry, Charité Universitätsmedizin, Berlin, Germany

**Keywords:** clinical high risk for psychosis (CHR-P), comorbidity, network analysis, attenuated psychotic symptoms, depressive symptoms, anxiety symptoms, general functioning

## Abstract

**Objective:**

Youths at clinical high risk for psychosis (CHR-P) are characterized by a high prevalence of anxiety and depressive disorders. The present study aimed at developing and analyzing a network structure of CHR-P symptom domains (i.e., positive, negative, disorganization, and general subclinical psychotic symptoms), depressive and anxiety symptoms, and general functioning.

**Methods:**

Network analysis was applied to data on 111 CHR-P children and adolescents (*M_*age*_* = 14.1), who were assessed using the Structured Interview for Prodromal Syndromes, the Children’s Depression Inventory, the Children’s Global Assessment Scale, and the Multidimensional Anxiety Scale for Children.

**Results:**

In the network, negative and disorganization symptoms showed the strongest association (*r* = 0.71), and depressive and anxiety symptoms showed dense within-domain connections, with a main bridging role played by physical symptoms of anxiety. The positive symptom cluster was not associated with any other node. The network stability coefficient (CS) was slightly below 0.25, and observed correlations observed ranged from 0.35 to 0.71.

**Conclusion:**

The lack of association between subclinical positive symptoms and other network variables confirmed the independent nature of subclinical positive symptoms from comorbid symptoms, which were found to play a central role in the analyzed network. Complex interventions should be developed to target positive and comorbid symptoms, prioritizing those with the most significant impact on functioning and the most relevance for the young individual, through a shared decision-making process. Importantly, the results suggest that negative and disorganization symptoms, as well as depressive and anxiety symptoms, may be targeted simultaneously.

## Introduction

Clinical high risk for psychosis (CHR-P) criteria are the most widely used criteria to identify individuals at imminent risk of developing a psychotic disorder. CHR-P criteria include a family history of schizophrenia (or a schizotypal personality disorder), a decline in social and occupational functioning, and the presence or worsening of attenuated psychotic symptoms (i.e., attenuated for frequency, duration, and intensity) (1–3). CHR-P individuals are mostly help-seeking adolescents or young adults who suffer from comorbid mental disorders, poor cognition, and poor social and occupational functioning; as a result, their quality of life is generally impaired (4–9). Moreover, their experience of subclinical psychotic symptoms may contribute to their withdrawal from complex and nuanced developmental tasks, leading to adverse outcomes (6, 10, 11). A longer duration of untreated subclinical psychotic symptoms (which typically onset approximately 1.85 years in advance of the CHR-P designation (12) is associated with poorer outcomes (6), a lower rate of remission, and a higher rate of transition to psychosis (13). Accordingly, preventive interventions are recommended as early as possible, with the goal of preventing, postponing, or ameliorating the onset of full-blown psychotic disorder (5, 12). Individuals who meet the CHR-P criteria present a cumulative transition risk of 0.15 at 1 year, 0.19 at 2 years, 0.25 at 3 years, 0.27 at 4 years, and 0.28 at more than 4 years (5), and tend to maintain symptoms and poor functioning for years (14, 15). Moreover, young people may never transition but present with chronic low functioning, fluctuating remission, recurrence and relapse of CHR-P status (6, 16–18).

Most research focuses on adult CHR-P individuals, whereas relatively little evidence has been conducted with samples of CHR-P children and adolescents (3, 19). A recent review and meta-analytical study on CHR-P adolescents (3) displayed the presence of comorbid disorders (i.e., anxiety and depressive disorders), impaired cognition and functioning–with transition rates of 10.4% at 6 months, 20% at 12 months, 23% at 24 months, and 25% at more than 36 months in underage CHR-P youth (3). Moreover, research evidence displayed that 60% of CHR-P adolescents did not recover and continued expressing symptoms after 6-year follow-up (3, 20); in addition, presenting before the age of 15 was associated with worse social functioning (3, 21). Finally, findings indicated that CHR-P adolescents showed more social stress (22), previous exposure to trauma (23, 24), and a higher likelihood of attempting suicide (25) than matched healthy controls (3).

Despite its utility for identifying individuals at imminent risk of psychosis, the CHR-P paradigm exhibits some limitations that merit further research. First, CHR-P individuals, at intake, show a large array of other full-fledged, clinically debilitating mental disorders, which can significantly hinder both treatment planning and the efficacy of interventions (14, 26–31). Specifically, meta-analytical evidence indicates that 73% of CHR-P individuals present at least one comorbid mental disorder, with 41 and 15% suffering from depressive and anxiety disorders, respectively (28). Also, the presenting complain CHR-P individuals seeks help for, may not be related to attenuated psychotic features and need to be considered actively (32). Depressive disorders, in turn, are related to negative symptoms and decreased remission from the CHR-P status, which may further impact general functioning (14, 28). Second, both the CHR-P diagnostic criteria and current preventive interventions are mainly focused on positive symptoms, while frequently neglecting other psychopathological domains. One of the exceptions is represented by the PACE clinic, which uses different modules to address symptomatology beyond attenuated psychotic features (18, 33). The efficacy of early interventions is therefore limited to ameliorating positive symptoms and preventing psychosis (34), whereas there is a lack of evidence for their longitudinal improvement of depressive and negative symptoms and general functioning (14, 35) (the step trial is an attempt to provide stepwise psychological and psychotropic interventions to address functioning overall as primary aim (36). Therefore, a crucial challenge is devising broader at-risk frameworks to capture early psychopathology manifestation and, at the same time, “maximize clinical utility” (37).

A promising framework for investigating CHR-P comorbidity and symptom co-occurrence is provided by the so-called network theory of mental disorders. At the core of this approach, there is an assumption that psychiatric symptoms, rather than representing passive manifestations of a common cause (as cancer cells are the common cause of oncological symptoms), actively contribute to sustaining each other (38). Thus, what we phenomenologically recognize as a mental disorder is considered a feedback loop among symptoms, leading to a state of prolonged symptom activation (39, 40). Accordingly, mental disorders are understood as a “stable state of strongly connected networks” (38). Moreover, network theory aligns with the evidence that: (a) psychopathology presents a multifactorial background, (b) several mechanisms that contribute to maintaining mental disorders are multidimensional, (c) pluralistic explanations are necessary in psychopathology (41–44). Moreover, it is suggested that mental disorders cannot be reduced only to a biological basis and that a holistic research strategy is required in this field (44). Moreover, the network’s relations depend also on mental state contents and cultural and historical features (44).

Various statistical network models have been developed to analyze the co-occurrence of symptoms estimated from data, using, for example, clinical interviews, and questionnaires for psychosis spectrum disorders, see (45–49). In these network models, *nodes* represent symptoms, and *edges* represent the unique associations between symptoms (50). Specifically, edge weight parameters (i.e., edges present a specific strength) denote the unique statistical associations (positive or negative) between two nodes, while controlling for the influence of all other nodes in the network (51). Furthermore, since some symptoms occur in multiple disorders, symptom activation can spread between syndromes, with the symptoms that bridge these syndromes playing a critical role in psychiatric comorbidity [i.e., the *comorbidity hypothesis* (40)]. Finally, to assess the relative importance of symptoms in psychopathology networks estimated from observational data, the concept of *node centrality* identifies nodes with a more central role in defining the network structure (52). Translating this statistical concept to psychology (40), the *centrality hypothesis* posits that the most central nodes are the best intervention targets, because they represent the most influential nodes (i.e., those holding the network, or the symptoms, together) in a given network structure (53). Therefore, centrality metrics are assessed to detect possible intervention targets (51, 54–57). Previous research employed network analysis to explore the interrelations of symptoms in samples of CHR-P, first-episode psychosis (FEP) and depressive individuals (47, 48), and CHR-P and healthy participants (58), highlighting the presence of sub-groups of symptoms and the relevance of the interplay between subclinical psychotic symptoms and comorbid non-psychotic symptoms, respectively.

Based on the above, the application of network theory to CHR-P might generate insight into the interrelations among psychotic and non-psychotic symptoms, and their causal interplay in the maintenance and spread of psychopathological manifestations, with potential clinical implications. The present study therefore applied network analysis to investigate the interrelations among depressive, anxiety, and attenuated psychotic symptoms (i.e., positive, negative, disorganization, and general symptoms) and general functioning in a clinical sample of CHR-P children and adolescents.

## Materials and methods

A total of 111 individuals meeting CHR-P criteria (*M_*age*_* = 14.1) were recruited from the Bambino Gesù Hospital in Rome. The numerosity was relatively small compared to other studies in the network field. The inclusion criteria were as follows: (a) the presence of a CHR-P condition according to the Structured Interview for Prodomal Syndromes (SIPS), (b) the absence of a full-blown psychotic disorder according to the *Diagnostic and Statistical Manual of Mental Disorders, Fifth Edition* (DSM-5) (59), (c) the absence of brain injury or neurological disease, (d) IQ > 70, (e) fluency in Italian, and (f) parental consent for participants younger than 18 years. Accordingly, participants had one or more CHR-P conditions (60) [i.e., attenuated positive symptom psychosis-risk syndrome (APS), brief intermittent psychosis psychosis-risk syndrome (BIPS), genetic risk and deterioration psychosis-risk syndrome (GRD)] with no full-blown psychotic disorder, and/or a psychotic symptoms (POPS) state, according to the SIPS (see section “Measures”). Participants were assessed by a psychiatrist or psychologist. The study obtained approval from the Ethics Committee of the Bambino Gesù Pediatric Hospital and the Ethics Committee of the Department of Dynamic and Clinical Psychology, Sapienza University of Rome (n°44/2017). Clinicians delivered written informed consent and withheld all identifying information about the participants. Table 1 reports the demographic and clinical characteristics of the participants.

**TABLE 1:** Demographics and clinical characteristics of participants.

	Sample (*N* = 111)
Age, years (*M*, *SD*)	14.1 (2.15)
Gender (m/f), *n*%	60 (54.1)/51 (45.9)
IQ *(M, SD)*	98.1 (15.9)
Years of education	8.76 (1.99)
Familiarity to psychiatric disorders, *n%*	47 (43.3)
**CHR-P type^a^, *n*%**	
• GRD	14 (12)
• APS	104 (93)
• BIPS	6 (5.4)
**Diagnosis, *n*%**	
• Specific learning disorder	3 (2.7)
• Cyclothymic disorder	1 (0.9)
• Disruptive mood dysregulation disorder	3 (2.7)
• Major depressive disorder	27 (24.3)
• Unspecified depressive disorder	2 (1.8)
• Specific phobia	1 (0.9)
• Social phobia	3 (2.7)
• Generalized anxiety disorder	14 (12.6)
• Obsessive compulsive disorder	24 (21.6)
• Post-traumatic stress disorder	2 (1.8)
• Unspecified feeding or eating disorder	3 (2.7)
• Enuresis	1 (0.9)
• Unspecified disruptive, impulse-control, and conduct disorder	7 (6.3)
• Personality disorder	2 (1.8)
**Symptoms *(M, SD)***	
• Positive symptoms	10.7 (3.8)
• Negative symptoms	19.9 (7.5)
• Disorganization symptoms	10.9 (5.1)
• General symptoms	11.5 (4.3)
**Medications, *n%***	
• Antidepressants	19 (17.1)
• Antipsychotics	33 (29.7)

^a^GRD, Genetic risk and deterioration psychosis-risk syndrome; APS, attenuated positive symptom psychosis-risk syndrome; BIPS, brief intermittent psychosis psychosis-risk syndrome.

### Measures

The *Structured Interview for Prodromal Syndromes* (SIPS) (61, 62) is a structured interview that is used to detect a CHR-P condition. It comprises four measures: (1) the Scale of Prodromal Symptoms (SOPS), (2) the DSM-IV Schizotypal Personality Disorder Checklist, (3) a questionnaire pertaining to family history of mental illness, and (4) the Global Assessment of Functioning scale. The SOPS assesses 19 prodromal symptoms clustered into four subscales: positive symptoms, negative symptoms, disorganization symptoms, and general symptoms. In each subscale, symptoms are rated from 0 (*never*) to 6 (*severe*). If at least one positive symptom is rated 3, 4, or 5, it means that the person meets the CHR-P criteria. Across studies, the median agreement for the CHR-P diagnosis as kappa (63) was 0.89 (range > 0.70–1.00), and the median reliability coefficient of the SOPS score was 0.90 (range > 0.75–0.96). Overall, the weight of the evidence supports the convergent and discriminant validity of the distinction between SIPS psychosis and CHR-P status (64).

The *Children’s Depression Inventory* (CDI) (65) is used to assess depressive symptoms in children and adolescents, though it also includes items aimed at evaluating scholastic and relational concerns. It is composed of 27 items, which are ranked from 0 to 2, providing a total score in the range of 0–54. Symptoms are clustered into three subscales: (1) negative mood, (2) negative self-esteem, and (3) interpersonal problems. Participants answer items with respect to how they have felt over the past 2 weeks. The measure showed high internal consistency (α = 0.80) and significant correlations between item and total product moment (66).

The *Multidimensional Anxiety Scale for Children* (MASC) (67) is a 39-item self-report instrument for assessing anxiety symptoms in children and adolescents. Items are clustered into four subscales: (1) physical symptoms, (2) social anxiety, (3) harm avoidance, and (4) separation anxiety. This measure showed a good internal consistency (α = 0.60 to α = 0.85) and a high test–retest reliability (*r* = 0.79 to *r* = 0.93) (68–70).

The *Children’s Global Assessment Scale* (CGAS) (71) is a measure used by clinicians to assess functioning in children and adolescents. It provides a total score for the level of disturbance in general functioning in the range of 0–100, with higher scores corresponding to higher levels of functioning. Studies demonstrated a fair to adequate inter-rater reliability (r = 0.53 to 0.87) (72–74).

### Network analysis

The interplay among psychopathology domains in a network structure was investigated using network analysis. In a network structure, symptoms are referred to as nodes, and the statistical relationships between two nodes are referred to as edges. In psychopathology networks, edges (i.e., relationships between symptoms) are not known *a priori*, but estimated from data on the basis of measured correlations. Since variables are not binary, a Gaussian graphical model (31) can be used to estimate the network structure. As this model does not take into account the directionality of the edges, it generates an undirected network structure. Accordingly, the model cannot provide insight into the direction of the relationships between nodes. However, it can reveal the presence of a feedback loop (i.e., circular correlations). Furthermore, the presence of an edge in the estimated network indicates a conditional dependence between the variables represented by the connected nodes (while controlling for all other variables). This dependence, expressed through partial correlations (the edge value), may underlie a causal relationship.

Since the numerosity of psychological data is rarely sufficient to enable the estimation of all network parameters (i.e., the number of parameters grows exponentially with the number of nodes), a regularization procedure is applied using the LASSO operator. This regularization shrinks all small correlations to zero, forcing the model to use only sufficiently strong edges to build the network structure. Hence, if a connection between two nodes is not present, it is not relevant for the considered network (i.e., not needed to explain the covariation structure).

To investigate the role of single symptoms in the network, we computed centrality indices for each node. Specifically, we focused on three indices: node strength (i.e., the degree to which a given node is directly connected to all other nodes), closeness (i.e., the degree to which a given node is indirectly connected to all other nodes), and betweenness (i.e., the weighted shortest paths between all pairs of nodes that pass through a given node) (75, 76). Furthermore, to assess the stability of centrality indices (i.e., the degree to which the indices remain robust within subsets of the data), we calculated the stability coefficient (CS). The CS is the maximum proportion of the dataset that can be dropped while maintaining a correlation of at least 0.7 between the recalculated indices and the indices of the original full sample. An acceptable CS is at least 0.25, and an excellent CS is at least 0.50. Our network analysis procedure was aligned with the relevant references in the field (38, 51, 75). Participants with missing data were not included in the sample. Analyses were conducted using the R software (version 4.2) [(77), and specifically the bootnet and qgraph packages].

## Results

**FIGURE 1:**
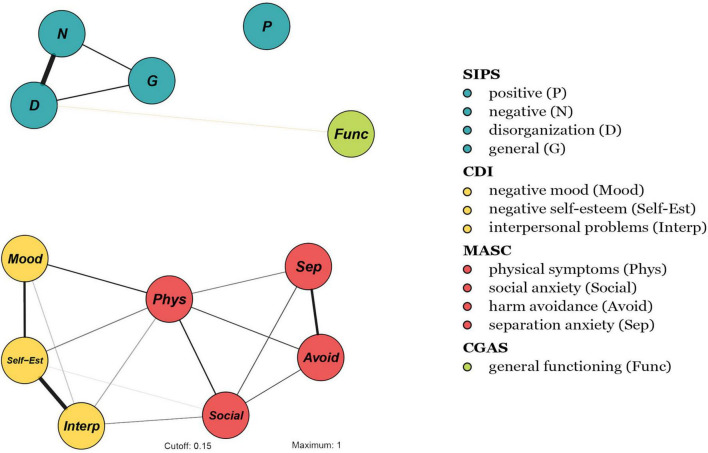
Network structure of 13 symptoms (based on symptomatology, as assessed with the SIPS, CDI, MASC, and CGAS). Node colors refer to *a priori* symptom domains (see legend) and numbers refer to specific individual items (i.e., symptoms) (see section “Measures”). The associations are either positive (colored black) or negative (colored red), with thicker lines representing stronger associations.

**FIGURE 2:**
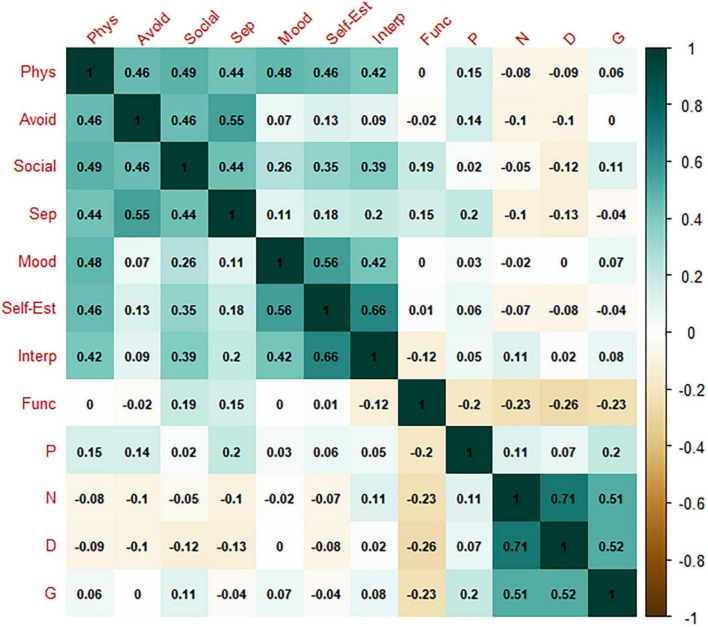
Correlation matrix. Phys, physical symptoms; Avoid, harm avoidance; Social, social anxiety; Sep, separation anxiety; Mood, negative mood; Self-Est, negative self-esteem; Interp, interpersonal problems; Func, general functioning; P, psychotic symptoms; N, negative symptoms; D, disorganization symptoms; G, general symptoms.

Figure 1 displays the network structure. For positive, negative, disorganization, and general symptoms, we refer to the subclinical psychotic symptoms, as evaluated by the SIPS. Not all symptoms were connected. In fact, *positive symptoms* did not exhibit any connection—either with other sub-threshold psychotic symptoms or with depressive and anxiety symptoms or general functioning. Notably, the attenuated psychotic symptom cluster was not linked to anxiety or depressive symptoms, through either direct or indirect edges. Moreover, as reported in Figure 2, in-cluster correlations were stronger than correlations across symptom domains. In this respect, the network structure exhibited three “archipelagos of symptoms” (38) that is, three separate subgraphs: (1) a component made of all of the considered depressive and anxiety symptoms, (2) a fully connected component (i.e., with all nodes connected) with attenuated psychotic symptoms (excluding positive symptoms) weakly linked to *general functioning*, and (3) a component comprised of only *positive symptoms*, represented by an isolated node. The most central nodes (i.e., the most influential nodes) were *physical symptoms* and *low self-esteem* in the first symptom sub-group and *disorganization* and *negative symptoms* in the second. The strongest correlation was between *negative symptoms* and *disorganization symptoms* (*r* = 0.71), whereas the only negative correlation was between *disorganization symptoms* and *general functioning* (*r* = 0.26), since the scales have opposite directions (i.e., general functioning deteriorates as disorganization symptoms increase). Moreover, visual inspection revealed that depressive and anxiety symptoms showed dense within-domain connections, indicating a bridging role played mainly by *physical symptoms*; in fact, *physical symptoms* were connected to all other anxiety and depressive symptoms, while, among the depressive symptoms, *social anxiety* was connected with *interpersonal problems* and *negative self-esteem.*

**FIGURE 3:**
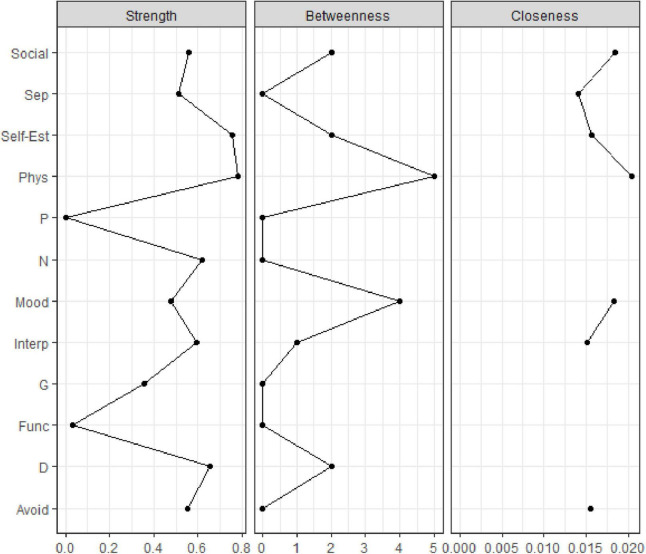
Centrality indices of the study variables within the network. Centrality indices (i.e., node strength, closeness, betweenness) are shown as standardized *z*-scores.

The correlation matrix is shown in Figure 2. Figure 3 plots the network centrality indices. The network CS was slightly below 0.25 (see Supplementary Figure 1). The bootstrapped confidence intervals of the estimated edge weights are reported in Supplementary Figure 2.

## Discussion

Since that network theory posits that symptom associations are not caused by a latent variable, but they are “real interactions,” comorbidity cannot be considered as a methodological problem, but “the substantiative flesh and bones of psychopathology” (40, 54, 78). Although meta-analytical research displayed the presence of poor functioning and debilitating comorbid mental disorders in CHR-P individuals (3, 4, 28), relatively little is known about their causal interplay and co-occurrence–which supported the adoption of the network theory insights and methods instead of other methodological approaches. Moreover, in understating and assessing emerging mental disorders, network theory as an organizing principle which embodies a more refined understating of how symptoms tend to engage in feedback loops (53, 78) may offer relevant insights into which symptom domains tend to co-occur and reinforce each other–ultimately, we consider that such a framework may inform preventive strategies aiming of limiting the spreading of early psychopathology manifestations.

The results confirmed a network structure embedding subclinical psychotic, depressive, and anxiety symptoms, as well as general functioning. Upon visual inspection, subclinical psychotic symptoms showed no association with non-psychotic symptoms, whereas depressive and anxiety symptoms were connected. Please remember that the spatial arrangement of the nodes in the network is not interpretable, and it should not be relevant for the conclusions.

The presence of a bridge symptom between anxiety and depression suggests a tendency for these symptoms to co-activate at this developmental stage (38). More specifically, *physical symptoms* were associated with a large array of depressive nodes. Interestingly, cognitive behavioral models distinguish between the cognitive and the physical components of anxiety disorders (79), pointing to the importance of an individual’s interpretation of physical experiences (80). Moreover, clinicians have reported that CHR-P youth tend to catastrophize their experiences and become hypervigilant (80). Therefore, according to the network structure, we could hypothesize that physical symptoms are associated with maladaptive beliefs, which, in turn, may co-activate depressive symptoms. Notably, although the network showed a co-occurrence between depressive and physical symptoms, it was not possible to establish a direction or causality of this relationship (51, 53).

Conversely, no bridge between subclinical psychotic symptoms and non-psychotic symptoms was detected. According to the network theory of mental disorders, an active symptom increases the likelihood that close symptoms in the network will be activated—in other words, strongly related symptoms tend to synchronize (38, 40). The absence of bridge symptoms between subclinical psychotic symptoms and non-psychotic symptoms seemed to reflect either distance in the network or different “archipelagos of symptoms.” Networks with limited connections (as in the present network) or weak connections are more likely to manifest an asymptomatic state than a closely interconnected network (38, 53). Importantly, the present sample was comprised of individuals who were younger (*M_*age*_* = 14.1) than those typically enrolled in CHR-P studies; thus, it is possible that the relatively weakly connected network (with no edge found even for general functioning) was sensitive to age, reflecting room for the prevention and remission of CHR-P status and other symptoms. Indeed, the typical age of psychosis onset is older than that of the present sample (81). Thus, early intervention in childhood and adolescence could have great preventive value, as the feedback loop among different psychopathology domains seems not yet fully structured and not self-sustaining (38, 47).

In the network, general functioning displayed a negative association with disorganization symptoms, in line with the results of prior research (82, 83). Moreover, one study found that individuals with poor social functioning, impaired processing speed, and high SOPS disorganization (>4) were almost five times more likely to experience poor social outcomes (83). Also, high general psychopathology has been reportedly associated with unfavorable outcomes (84). However, in the present study, this link was one of the weakest. Thus, further research is needed to outline the potential longitudinal interplay between general functioning and other nodes.

Regarding the psychotic psychopathological domain, the strongest association (i.e., edge) was between negative and disorganization symptoms. Although negative symptoms (i.e., avolition, anhedonia, abulia, blunted affect) have been relatively neglected and understudied (mainly for cultural reasons (85–87), they are clinically relevant (10, 88, 89), as longitudinal data indicate that 80% of CHR-P individuals suffer from at least one moderate or severe negative symptom at intake (90). Velligan et al. (91) hypothesized a negative symptoms feedback loop in which withdrawal leads to decreased initiative and interest, and eventually atrophy in role functioning and social skills, fostering the expression of negative symptoms. In addition, experience of early subclinical symptoms may exacerbate feelings of alienation and social isolation (91–93). Focusing on the relationship between negative and disorganization symptoms in the network structure, an explanatory framework may be offered by the aberrant salience model, which posits that dysregulation of the dopamine system may lead to inappropriate attention to irrelevant stimuli (58, 94, 95). Indeed, such dysregulation has been linked to negative symptoms (e.g., anhedonia, blunted affect), which, in turn, have been connected to cognitive deficits and disorganization (96–98). Moreover, in the aberrant salience model, positive psychotic symptoms are hypothesized to emerge after the dysregulation of the dopamine system, to make sense of the perceived world (which would otherwise appear unintelligible) (58, 95, 99).

Alternatively, we suggest that the finding of a strong association (i.e., edge) between negative and disorganization symptoms could be explained by a third latent variable not considered in the present study: self-disorders. The phenomenological ipseity-disturbance model views self-disorders as the pathogenic core of schizophrenia spectrum disorders (86). Self-disorders can be depicted as long-lasting non-psychotic experiences and distortions in subjectivity (100–102). A recent study found that anomalous self-experiences were strongly related to both negative and disorganization symptoms in a sample of CHR-P individuals, whereas they exhibited a weaker relation with positive symptoms (103). Nevertheless, since other research (104–106) points out that self-disorders may also show relevant associations with subclinical positive symptoms (which, in our network, were not connected to other nodes), further research is needed to test the mediating role of self-disorders.

These explanations are aligned with the network structure found in the present study, in which positive symptoms had no association with any other symptom in the network. This is of particular interest, since the results of the network analysis suggest that the main target of current preventive interventions (i.e., positive symptoms) does not represent a “core” node or have meaningful connections with other symptom domains. Indeed, a recent study estimating age-specific (i.e., ages 9–17 years vs. ages 18–45 years) prediction models of psychosis (107) showed that the conversion outcome was best predicted by negative symptoms in CHR-P adolescents, but positive symptoms in CHR-P adults (107). The lack of associations of positive symptoms with other nodes it is partially in line with a recent study informed (108) by the Hierarchical Taxonomy of Psychopathology [HiTOP (109, 110)]. In this study (108), an explanatory factor analysis in a CHR-P group was employed, showing three different psychopathology dimensions: internalizing, primarily negative symptoms, and primarily positive symptoms. If, on the one hand, the three archipelagos of symptoms of our network and the three dimensions of such research present several similarities, on the other, there are some differences. We can list, for example, the role of depression, which loaded onto internalizing and negative symptoms in this previous study mentioned above. Conversely, we did not find a relevant connection between depressive and negative symptoms. The authors (108) emphasized the need for further research on the “dividing lines” between depressive and negative symptoms. Moreover, the authors found small to medium factor intercorrelations, whereas we did not find relevant associations among symptom sub-groups. Despite the theoretical differences between the network theory and the HiTOP, both models supported different ways in which properties tend to hang together in CHR-P samples.

According to the network theory of mental disorders, the benefits of any clinical intervention would not be expected to spread to other network nodes unless its main targets were core symptoms or relevant bridge symptoms (38, 47). Accordingly, interventions for anxiety and depression may not be effective for psychotic symptoms (and vice versa), in children and adolescents. However, other symptoms that were not considered in the present network analysis might benefit from universal prevention strategies (111).

Regarding medications, meta-analytical evidence shows that about a quarter of CHR-P individuals has already been exposed to antipsychotics (AP) at the time of CHR-P status ascription (106)–the administration of APs in our sample aligns with this notion. In this study, APs administration was associated with an increased risk of imminent conversion to psychosis; a possible explanation is that APs prescription may be a “red flag,” an indicator of a greater baseline severity that needs to be carefully monitored (112). Moreover, recent meta-analytical evidence with CHR-P individuals (113) displayed that being already exposed to antidepressants (ADs) treatment at intake was associated with a lower conversion rate to psychosis at follow-up. In discussing this result, it is relevant to consider that conversion to psychosis is based entirely on the progression of positive symptoms. As a result, it is possible that depressive symptoms may interact over time with positive symptoms since ADs were associated with a lower risk of conversion to psychosis in the aforementioned meta-analytical study. This may suggest that despite relevant connections–either direct or indirect–between depressive symptoms and positive symptoms were not observed in our “baseline” network, they may indeed emerge over time. Consequently, depressive symptoms should not be dismissed in preventive intervention strategies. Moreover, it is possible that our network structure may have been partially influenced by the APs and ADs administration.

Moreover, there is an ongoing debate about the possibility of planning intervention strategies not exclusively focused on the reduction of the positive symptoms and preventing transition to psychosis, but also on the improvement of both functional outcome and quality of life, as well as on the reduction of comorbidity rates (11, 36, 37)–which “need full consideration” (114). Moreover, transdiagnostic clinical staging models have been proposed (37, 115), with the general aims of broadening the detection of risk beyond psychosis (i.e., including different outcome syndromes and considering different potential homotypic and heterotypic illness trajectories) and improving the clinical utility of early detection and intervention (37).

The present results should be read in light of some study limitations. First, the network CS was not excellent, likely due to the relatively small study sample; thus, caution is warranted in the interpretation of the centrality measures. Nevertheless, since it is recommended that centrality differences only be interpreted when CS > 0.25 (51), our analysis focused mainly on relevant edges. Since psychopathology research tend to present limited sample sizes, network parameters may not be estimated precisely–and they may not approximate to the true value (51)–, and concerns about the replicability of network structures arise. Moreover, smaller samples and single-item assessments may negatively impact network stability (53, 116). To address this issue, methods to estimate the stability properties of the network have been developed as the CS value (51). Nevertheless, the replicability of network analysis still represents a debated topic (116, 117). Moreover, it has been debated that network built on cross-sectional data cannot be assumed to represent a generalization of the level of the single individual (53, 118–122). On the other hand, such cross-sectional networks have the potential to offer more refined information about symptom co-occurrence in samples with specific clinical characteristics (e.g., CHR-P individuals). Second, the cross-sectional nature of the study design did not allow us to investigate symptom progression; thus, prognostic hypotheses should also be made with caution. Nevertheless, a dimensional assessment of CHR-P status at intake may suggest treatment targets (i.e., specific symptoms) to prevent the establishment of bridge symptoms among different psychopathology domains over time (47, 50, 58). Finally, the results are inevitably measure-sensitive for example, potential other relevant connections would have been detected by employing the Comprehensive Assessment of At Risk Mental States (CAARMS) (1) instead of the SIPS.

## Data availability statement

The datasets presented in this article are not readily available because the dataset includes sensitive information, the corresponding author is available to provide anonymous data under request. Requests to access the datasets should be directed to corresponding author.

## Ethics statement

The studies involving human participants were reviewed and approved by the Ethics Committee of the Bambino Gesù Pediatric Hospital and the Ethics Committee of the Department of Dynamic and Clinical Psychology, Sapienza University of Rome. Written informed consent to participate in this study was provided by the participants or their legal guardian/next of kin.

## Author contributions

GLB: writing—original draft preparation and reviewing and editing. MP: funding acquisition and resources. EC: formal analysis, visualization, and writing—reviewing and editing. AP and ASL: writing—reviewing and editing. SV and VL: supervision. TB: conceptualization, project administration, and writing—reviewing and editing. MS: conceptualization, methodology, writing—reviewing and editing, data curation, and supervision. All authors contributed to the article and approved the submitted version.
