# Supplementary material for: A network analysis of anxiety, depressive, and psychotic symptoms and functioning in children and adolescents at clinical high risk for psychosis
Source: Front Psychiatry. 2022 Oct 28;13:1016154. doi: 10.3389/fpsyt.2022.1016154 (PMC9650363; doi:10.3389/fpsyt.2022.1016154)
Supplement: Supplementary file 1 [file Data_Sheet_1.docx]

**Supplementary materials on**

**A Network Analysis of Anxiety, Depressive, and Psychotic Symptoms and Functioning in Children and Adolescents at Clinical High Risk for Psychosis.**

**Gabriele Lo Buglio^1^, Maria Pontillo^2^, Erika Cerasti^1,3^, Andrea Polari^4,5^, Arianna Schiano Lomoriello^6^, Stefano Vicari^2,7^, Vittorio Lingiardi^1^, Tommaso Boldrini^8*^**^†^**, Marco Solmi^,9,10,11,12,13†^**

†These authors contributed equally to this work and share last authorship

^1^Department of Dynamic and Clinical Psychology, and Health Studies, Faculty of Medicine and Psychology, Sapienza University of Rome, Rome, Italy

^2^Child Psychiatry Unit, Department of Neuroscience Bambino Gesù Children's Hospital, IRCCS Rome, Italy

^3^ Istat Istituto Nazionale di Statistica, Rome, Italy

^4^Orygen Specialist Programs, Melbourne, Australia

^5^Centre for Youth Mental Health, The University of Melbourne, Parkville, Victoria, Australia

^6^Section for Cognitive Systems, DTU Compute, Technical University of Denmark, Kgs. Lyngby, Denmark

^7^Department of Life Science and Public Health, Catholic University of the Sacred Heart, Rome, Italy

^8^Department of Developmental Psychology and Socialization, University of Padova, Padova, Italy

^9^School of Epidemiology and Public Health, Faculty of Medicine, University of Ottawa, Ottawa, Canada

^10^Department of Psychiatry, University of Ottawa, Ontario, Canada

^11^Department of Mental Health, The Ottawa Hospital, Ontario, Canada

^12^Ottawa Hospital Research Institute (OHRI) Clinical Epidemiology Program University of Ottawa, Ottawa, Ontario

^13^Department of Child and Adolescent Psychiatry, Charité Universitätsmedizin, Berlin, Germany

***** Department of Developmental Psychology and Socialization, University of Padua, Via Venezia, 8, 35131, Padua, Italy; e-mail: tommaso.boldrini@unipd.it

Index

**Figure S1.** Average correlation between centrality indices of network subsamples and the original sample.

**Figure S2.** Bootstrapped confidence intervals of estimated edge weights for the network.


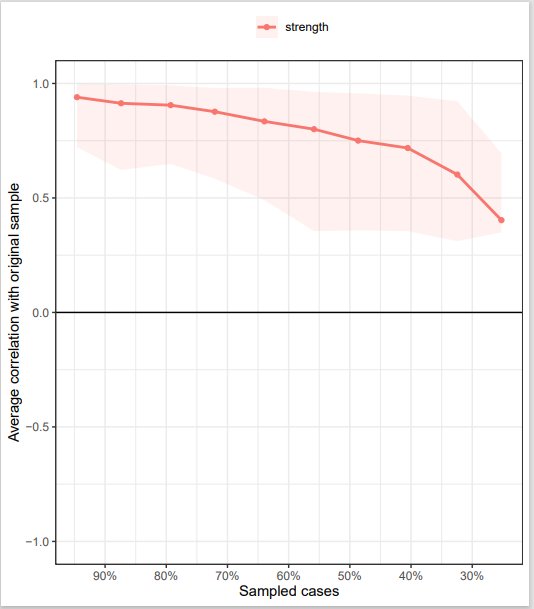


**Figure S1.** Average correlation between centrality indices of network subsamples and the original sample. Lines indicate means and areas indicate the range from the 2.5th quantile to the 97.5th quantile.


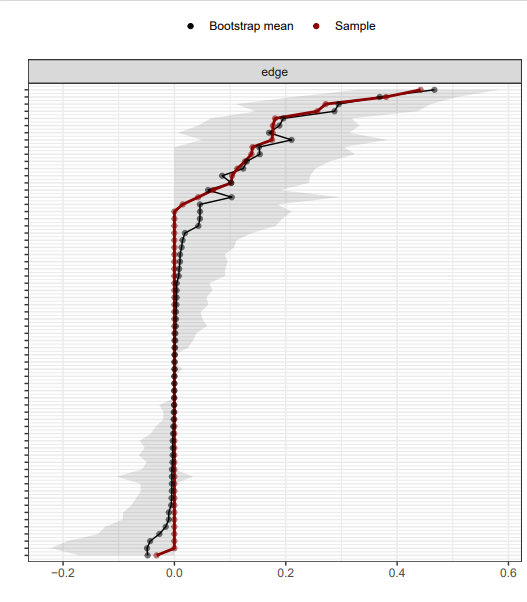


**Figure S2.** Bootstrapped confidence intervals of estimated edge weights for the network.
